# Increased Neurofilament Light Chain Blood Levels in Neurodegenerative Neurological Diseases

**DOI:** 10.1371/journal.pone.0075091

**Published:** 2013-09-20

**Authors:** Johanna Gaiottino, Niklas Norgren, Ruth Dobson, Joanne Topping, Ahuva Nissim, Andrea Malaspina, Jonathan P. Bestwick, Andreas U. Monsch, Axel Regeniter, Raija L. Lindberg, Ludwig Kappos, David Leppert, Axel Petzold, Gavin Giovannoni, Jens Kuhle

**Affiliations:** 1 Blizard Institute, Barts and the London School of Medicine and Dentistry, Queen Mary University of London, London, United Kingdom; 2 Bone & Joint Research Unit, John Vane Science Centre, Queen Mary University of London, London, United Kingdom; 3 UmanDiagnostics, Umeå, Sweden; 4 North-East London and Essex Regional MND Care Centre, London, United Kingdom; 5 Wolfson Institute of Preventive Medicine, Barts and The London School of Medicine and Dentistry, Queen Mary University of London, London, United Kingdom; 6 Memory Clinic, Department of Geriatrics, University Hospital, Basel, Basel, Switzerland; 7 Laboratory Medicine, University Hospital, Basel, Basel, Switzerland; 8 Department of Neurology, University Hospital, Basel, Basel, Switzerland; 9 UCL Institute of Neurology, Department of Neuroinflammation, London, United Kingdom; Innsbruck Medical University, Austria

## Abstract

**Objective:**

Neuronal damage is the morphological substrate of persisting neurological disability. Neurofilaments (Nf) are cytoskeletal proteins of neurons and their release into cerebrospinal fluid has shown encouraging results as a biomarker for neurodegeneration. This study aimed to validate the quantification of the Nf light chain (NfL) in blood samples, as a biofluid source easily accessible for longitudinal studies.

**Methods:**

We developed and applied a highly sensitive electrochemiluminescence (ECL) based immunoassay for quantification of NfL in blood and CSF.

**Results:**

Patients with Alzheimer’s disease (AD) (30.8 pg/ml, n=20), Guillain-Barré-syndrome (GBS) (79.4 pg/ml, n=19) or amyotrophic lateral sclerosis (ALS) (95.4 pg/ml, n=46) had higher serum NfL values than a control group of neurological patients without evidence of structural CNS damage (control patients, CP) (4.4 pg/ml, n=68, p<0.0001 for each comparison, p=0.002 for AD patients) and healthy controls (HC) (3.3 pg/ml, n=67, p<0.0001). Similar differences were seen in corresponding CSF samples. CSF and serum levels correlated in AD (r=0.48, p=0.033), GBS (r=0.79, p<0.0001) and ALS (r=0.70, p<0.0001), but not in CP (r=0.11, p=0.3739). The sensitivity and specificity of serum NfL for separating ALS from healthy controls was 91.3% and 91.0%.

**Conclusions:**

We developed and validated a novel ECL based sandwich immunoassay for the NfL protein in serum (NfL^Umea47:3^); levels in ALS were more than 20-fold higher than in controls. Our data supports further longitudinal studies of serum NfL in neurodegenerative diseases as a potential biomarker of on-going disease progression, and as a potential surrogate to quantify effects of neuroprotective drugs in clinical trials.

## Introduction

Neurofilaments (Nf) are highly specific major structural proteins of neurons, consisting predominantly of four subunits: Nf light (NfL), Nf medium (NfM) and Nf heavy (NfH) chain and alpha-internexin [1]. Nf are released in significant quantity following axonal damage or neuronal degeneration. Disruption to the axonal membrane releases Nf into the interstitial fluid and eventually into cerebrospinal fluid (CSF) and blood. Therefore, blood Nf levels could be useful for both predicting and monitoring disease progression and for assessing the efficacy and/or toxicity of future neuroprotective treatment strategies.

Several previous studies have demonstrated the presence of NfH and NfL in CSF, which has been assumed to reflect brain pathology more accurately than the peripheral blood compartment [2–12]. However, obtaining longitudinal CSF samples is considered too invasive outside the clinical trial arena, precluding the broader clinical use of Nf. In contrast to CSF, serial blood samples can readily be collected, hence reliable quantification of NfL in blood would be a major stride towards a biomarker of the course of neurodegeneration. Several reports have suggested peripheral blood levels of NfH as a potential marker of neurodegeneration [13–22]. In contrast to this, there is only one recent study investigating serum NfL; this paper examined the relationship between serum NfL and neurological outcome following cardiac arrest [23].

A commercially available ELISA (UmanDiagnostics NF-light^®^ assay) uses two highly specific, non-competing monoclonal antibodies (47:3 and 2:1) to quantify soluble NfL in CSF samples but it cannot in its present form be used for analysis of blood samples [24].

We recently compared our highly sensitive electrochemiluminescence (ECL)-based solid-phase sandwich immunoassay for NfH^SMI35^ (adhering to a previously proposed nomenclature, the soluble fraction of NfH measured is indicated with the capture antibody in the superscript [8]) with NfL, determined by the NF-light^®^ assay in CSF samples [6]. Importantly, the conventional ELISA showed higher sensitivity compared with the ECL-NfH^SMI35^ immunoassay [25].

The aim of this study was to develop and validate (both analytically and clinically) a sensitive ECL-based NfL assay suitable for the quantification of NfL in serum at concentrations relevant to clinical settings.

## Materials and Methods

### Antibodies and chemicals

The following mouse antibodies were used: Capture monoclonal antibody (mAB) 47:3, and the biotinylated detector mAB 2:1 [10,24]. MSD SULFO-TAG^TM^ labelled streptavidin was used as detection reagent to generate electrochemiluminescence (MSD, Gaithersburg, MD). Bovine serum albumin (BSA), ethylenediaminetetraacetic disodium salt (EDTA), NaCl, phosphate buffered saline, pH 7.5 (PBS), tris base and Tween 20 were of analytical grade (Sigma-Aldrich, Saint Louis, MO).

### Standards

Bovine lyophilized NfL was obtained from UmanDiagnostics (N Norgren). Standards were diluted in tris buffered saline (TBS) containing 1% BSA, 0.1% Tween 20, pH 7.5 and ranged from 0 to 10,000 pg/ml. Batch prepared standards were stored at -80°C.

### Patients and Control persons and ethics statement

Written informed consent was obtained from all patients in accordance with the Declaration of Helsinki, and the study was approved by the Common Institutional Review Board of the Cantons of Basel. Paired CSF and serum samples were collected during routine diagnostic investigations as indicated by the treating physicians.

Samples were collected and processed at room temperature within two hours. Serum samples were spun at 2,000 g, CSF samples at 400 g at room temperature for 10 minutes, aliquoted in polypropylene tubes and stored at -80°C.

Serum samples from 67 healthy control subjects (HC) were included in the study. For ethical reasons CSF samples were not available from these subjects. The group of control patients (CP) (n=68) consisted of patients who, based on extensive diagnostic evaluation had no objective clinical, structural (cranial magnetic resonance imaging, MRI), laboratory (CSF analysis) or functional (electroencephalography, EEG) deficit. These patients suffered from tension type headache (n=21), lower back pain (n=7), psychiatric disorders (n=26) or miscellaneous non-specific symptoms for which no neurological explanation could be found (n=14). From two of these patients there was not enough CSF left for further analysis. In addition, 49 patients with probable or definite ALS (for three no serum and for one no CSF sample was available) [26], probable Alzheimer’s disease (AD) [27], or a Guillain-Barré syndrome (GBS) (for one no serum sample was available) (n=20 each) were included (Table 1). The analyst was blinded to all clinical data.

**Table 1:** Demographic characteristics of healthy controls, control patients and patients.

	**HC**	**CP**	**AD**	**GBS**	**ALS**
**N**	67	68	20	20	49
**Females** (n [%])^a^	38 (56.7)	41 (60.3)	13 (65.0)	10 (50.0)	14 (28.6)
**Age** [years, median (IQR)]^b^	35.0 (28.0-42.0)	38.3 (27.5-46.4)	72.5 (70.1-80.2)	59.6 (39.1-71.7)	62.7 (54.5-70.7)

^a^There were less female ALS patients compared to HC (p=0.004), CP (p=0.001) and AD (p=0.007).

^b^HC and CP, were younger compared to AD, GBS and ALS (p<0.0001, respectively). HC: Healthy controls; CP: Control patients; AD: Alzheimer’s disease; GBS: Guillain-Barré syndrome; ALS: Amyotrophic lateral sclerosis; IQR: Interquartile range.

### Analytical procedure

The 96-well plates (Multi-Array^®^ plates, Meso Scale Discovery, Gaithersburg, MD) include integrated screen-printed carbon ink electrodes on the bottom of the wells. Coating was done overnight with 30 µl of capture antibody (mAB 47:3,1.25 µg/ml) diluted in PBS (pH 7.4) at 4°C. All following incubation steps were done on a plate shaker (800 rpm) and were preceded by three wash steps with 200 µl of TBS, containing 0.1% Tween 20 (pH 7.5) per well. Non-specific binding sites were blocked with 100 µl of TBS, containing 3% BSA, per well for 1h. After washing, 25 µl of TBS containing 1% BSA and 0.1% Tween 20 was added as sample diluent to each well. 25 µl of standard, control or serum/CSF sample was then added in duplicate and the plate incubated at room temperature (RT) for 2h. After washing, 25 µl of the secondary antibody (mAB 2:1, 0.5 µg/ml) diluted in TBS containing 1% BSA and 0.1% Tween 20 was added to each well and the plate incubated for 1 h at RT. After washing, MSD SULFO-TAG^TM^ labelled streptavidin (0.25 µg/ml), diluted in TBS containing 1% BSA and 0.1% Tween 20, was added and incubated for 1h at RT. Following a final wash, 150 µl of ECL read buffer (MSD) diluted 1:2 with distilled water was added and the ECL signal, detected by photodetectors, measured using the MSD Sector Imager 2400 plate reader. A four-parameter weighted logistic fit curve was generated, sample concentrations extrapolated and analysed using the Discovery Workbench 3.0 software (MSD). If required, samples were appropriately diluted to fall in the range of the standard curve. Non measurable NfL samples were reported as 0 pg/ml.

### Statistical analysis

Continuous variables were described by their median and interquartile range (IQR), and categorical variables by numbers and percentages. Comparison of demographic data was performed using the Kruskal-Wallis test, and pairwise post-hoc comparisons using Dunn’s post-test or chi-square test as appropriate. Serum and CSF levels of NfL were log-transformed to achieve a normal distribution for subsequent analysis. To control for age as a potential confounding factor, an analysis of covariance with age as covariate and disease group as fixed factor, was performed [7]. Group-specific levels of NfL were expressed as geometric means with 95%-confidence intervals. For log-normal variables, the geometric mean equals the median. Correlations were computed by determining the Spearman rank correlation coefficient (r). The cut-off (upper reference range of normal) providing optimal sensitivity and specificity in distinguishing ALS from HC by serum NfL was defined by receiver operating characteristic (ROC) curve analysis. Proportions above and below this cut-off were compared with the Chi-Square test. A two-sided p-value < 0.05 was considered as significant. P-values of post-hoc comparisons were adjusted using a Bonferroni correction. All statistical analyses and graphs were performed using SPSS (Version 15.0 SPSS, Chicago, IL) and Graph Pad Prism 5.02 for Windows (GraphPad Software, San Diego, CA).

## Results

### Reproducibility of the standard curve

**Figure 1:**
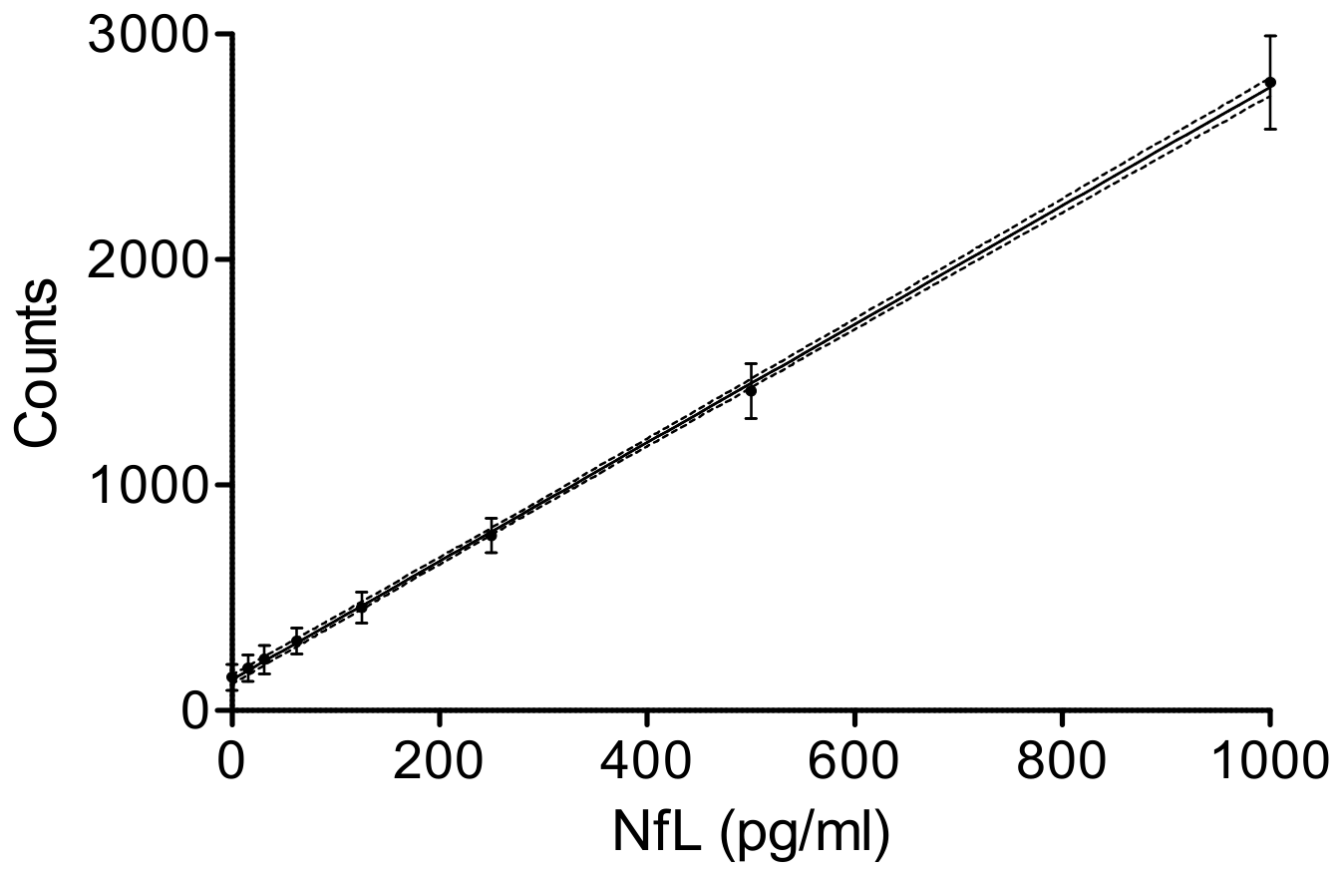
Reproducibility of the standard curve. Reproducibility of 20 consecutive standard curves. The graph shows the mean counts (dots) ± SD (bars), linear regression line and 5% and 95% confidence interval curves (broken lines) (R^2^=0.99).

Figure 1 shows the mean raw counts of 20 consecutive standard curves in the range of 0-1,000 pg/ml and the resulting regression line. Individual standard curves showed a high degree of linearity (R^2^ = 0.99) (Figure 1).

### Precision and accuracy

Reproducibility (intra-assay variability) and repeatability (inter-assay variability) of the assay was evaluated with native serum samples in 10 consecutive assays on independent days. In four independent samples of native serum the mean coefficients of variation (CV) of duplicates (intra-assay precision) for NfL^Umea47:3^ were 4.9% (12.1 pg/ml, sample 1), 5.5% (39.6 pg/ml, sample 2), 4.1% (83.1 pg/ml, sample 3) and 3.8% (103 pg/ml, sample 4, average: 4.6%). In CSF the mean intra-assay CVs were 6.0% (569 pg/ml, sample 1), 6.4% (3,645 pg/ml, sample 2), 2.7% (7,501 pg/ml, sample 3) and 6.8% (12,762 pg/ml, sample 4) averaging at 5.5%. Inter-assay CVs for serum were 23.6% (sample 1), 16.9% (sample 2), 8.5% (sample 3), and 10.9% (sample 4, average: 15.0%). In CSF inter-assay CVs were 10.3% (sample 1), 10.4% (sample 2), 6.7% (sample 3) and 11.7% (sample 4, average: 9.8%).

Recovery rates were tested in 6 serum samples from healthy volunteers. Recovery of NfL (serum spiked with 50 pg/ml of HPLC purified bovine NfL) was 72% and 114%. For serum spiked with 100 pg/ml of NfL it was 81% and 96%, and for 1,000 pg/ml of NfL recovery was 82% and 116%.

### Analytical sensitivity and stability of the analyte

Sensitivity (lowest standard above blank) was calculated as blank signal plus three standard deviations (SD) from 32 assays. The mean blank signal was 138 counts (SD 20.9 counts). The mean signal of the lowest standard (15.6 pg/ml) was 184.5 counts (SD 23.2): accordingly analytical sensitivity was defined to be 15.6 pg/ml. We tested the stability of NfL at room temperature (RT), 4 °C and compared this to samples stored at -80 °C. Four aliquoted serum samples were frozen at -80 °C. The aliquots were thawed on days 0, 3 hours before measurement, days 1, 4 and 8 and stored at RT or 4 °C until analysis. The measured signals were normalised to the signal of the day 0. There was no significant change in signal in samples stored at RT and at 4 °C (RT: day 8: 1.06 ± 0.08 (mean ± SD), p = 0.4063 and 4 °C: day 8: 1.01 ± 0.09, p = 0.1721). Four serum samples were analysed for stability during freeze-thawing cycles. The samples underwent 1, 2, 3, 4 or 5 freeze-thawing cycles and the signal was normalised to the sample freeze-thawed once, without any relevant effect of freeze-thawing on the measured signals (5 freeze-thawing cycles: 1.03 ± 0.03, p = 0.5076).

### Parallelism

**Figure 2:**
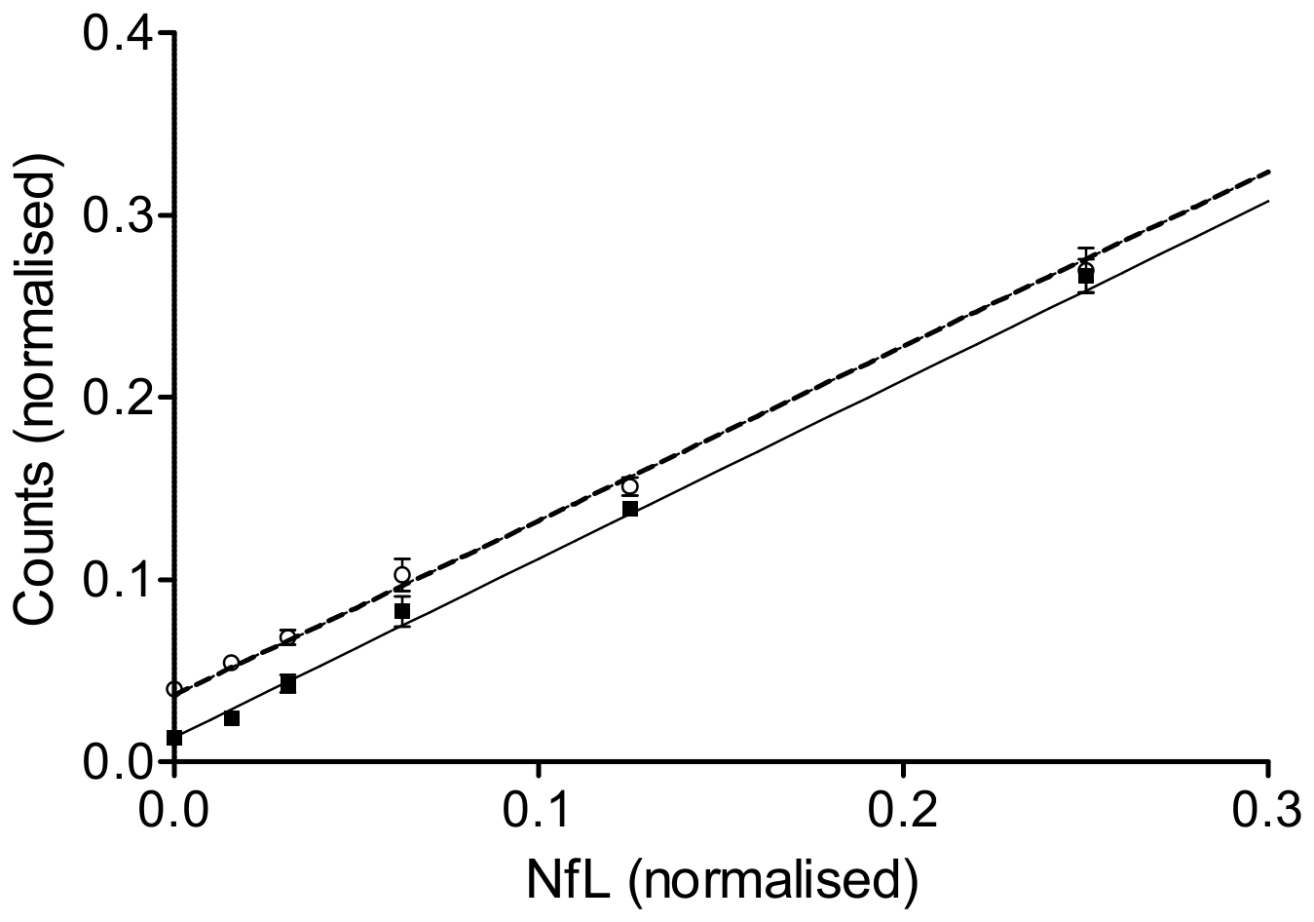
Parallelism between standards and serum dilutions. Parallelism for NfL between standards (open line, open dots) and serum (closed line, black squares). The linear regression lines, mean (open dots or black squares) and ±SD are shown.

We studied parallelism between standards and samples by reciprocal dilutions of three serum samples and three standard curves. The obtained signals were normalised to the highest value within this series (100%). The parallel relationship is demonstrated in Figure 2, suggesting the absence of aggregate formation or endogenous binding between NfL and other blood substrates [28] (Figure 2).

### Reference populations

NfL was determined in serum of 67 HC (56.7% females, median age 35.0 years) and in serum of 68 and CSF of 66 CP (60.3% female, median age: 38.3 years) (Table 1). Serum levels between HC (3.3 pg/ml, 2.0-5.3) and CP (4.4 pg/ml, 2.4-8.1) did not differ (p=1.0) and did not correlate with either age or gender. Conversely, CSF levels in CP correlated with age (r=0.68, p<0.0001).

### Neurological disease population

#### A. Serum

**Figure 3:**
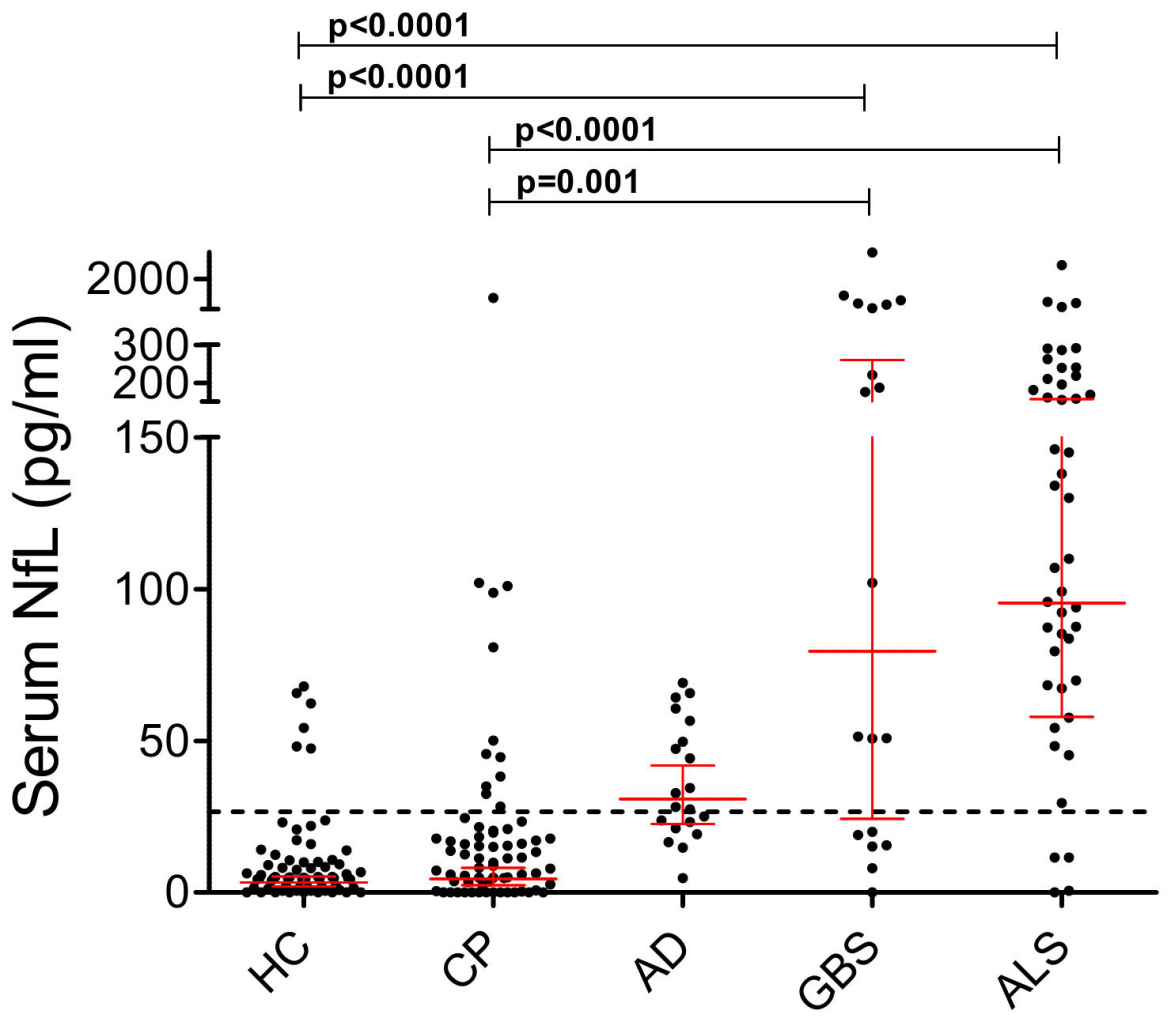
Serum NfL levels in the two reference groups (healthy controls, HC and control patients, CP) and neurological disease groups. Patients with a Guillain-Barré syndrome (GBS) (79.4 pg/ml) or Amyotrophic lateral sclerosis (ALS) (95.4 pg/ml) had higher values compared with HC (3.3 pg/ml; p<0.0001, respectively) and CP (4.4 pg/ml, p<0.0001 and p=0.001). Significances for comparisons between patients with Alzheimer’s disease (AD) and HC (p<0.0001) and AD and CP (p=0.002) were lost after age corrections. The horizontal dotted line represents the upper reference range (cut-off value) of 26.6 pg/ml. Geometric mean and 95% CI are displayed. Dots represent individual samples. P-values are adjusted for age and corrected by Bonferroni method.

AD (30.8 pg/ml, 22.6-41.9), GBS (79.4 pg/ml, 24.3-259.6) and ALS (95.4 pg/ml, 57.9-157.0) had higher serum NfL levels compared with HC and CP (AD versus CP: p=0.002 all other comparisons versus HC and CP: p<0.0001). NfL levels correlated with age in GBS (r=0.48, p=0.038) and ALS (r=0.30, p=0.04). In the age-corrected group comparisons versus HC and CP differences remained significant, except for the comparisons against AD (Figure 3).

A cut-off level of 26.6 pg/ml (Figure 3) for serum NfL resulted in a sensitivity of 91.3% and a specificity of 91.0% for differentiating ALS versus HC. A higher proportion (p<0.0001 for all comparisons) of patients had serum NfL values above this cut-off: 16/20 (80.0%) in AD, 13/19 (68.4%) in GBS, 42/46 (91.3%) in ALS, compared to HC (6/67, 9.0%).

#### B. CSF

NfL levels in AD (1396 pg/ml, 1139-1711), GBS (1361 pg/ml, 726-2554) and ALS (5513 pg/ml, 4151-7323) were higher than in CP (324 pg/ml, 282-372, p<0.0001 for all), and CSF NfL concentrations in ALS were higher than in AD and GBS (p<0.0001, respectively).

**Figure 4:**
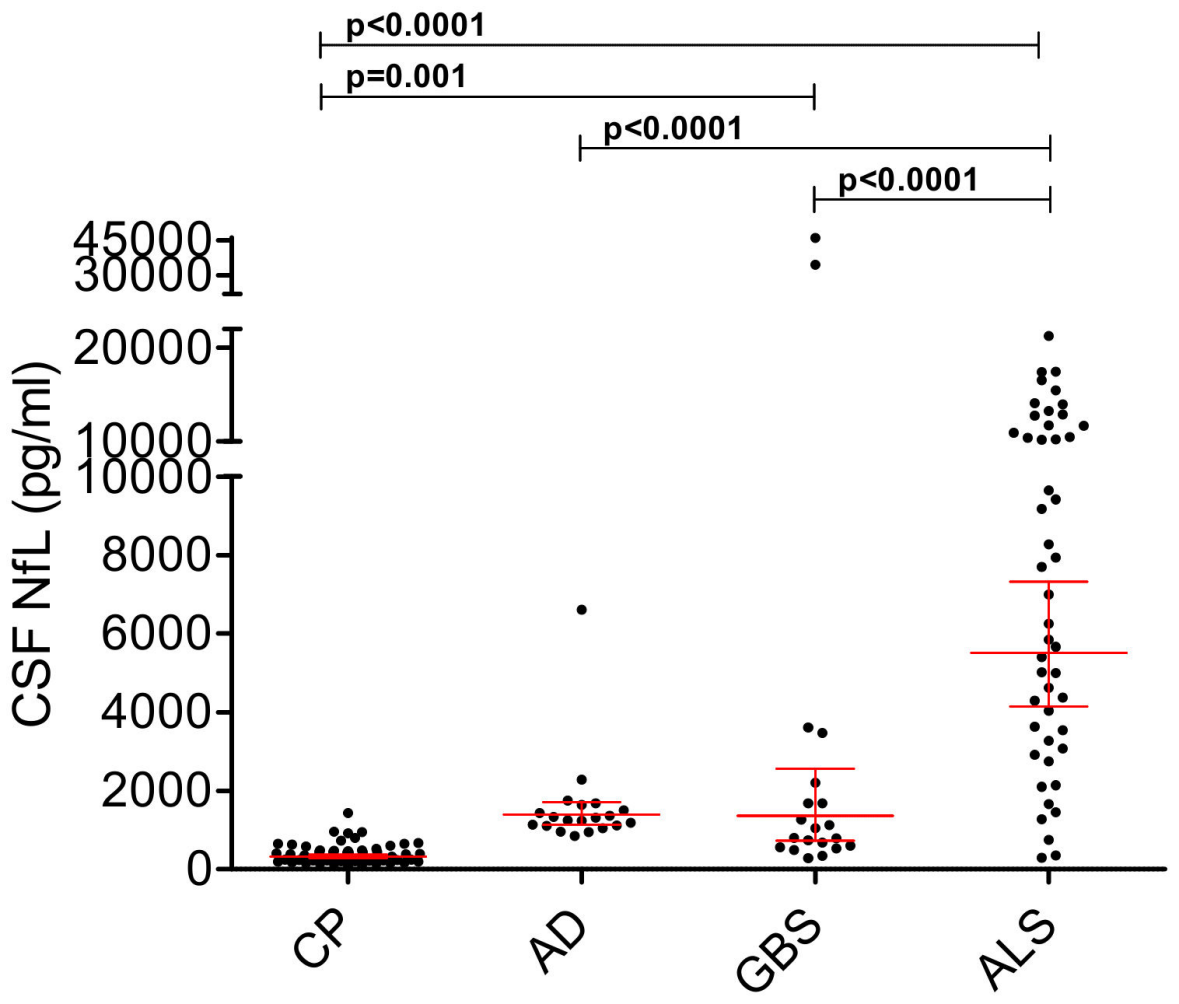
CSF NfL levels in the reference group (control patients, CP) and neurological disease cohorts. Patients with Amyotrophic lateral sclerosis (ALS) (5513 pg/ml) or a Guillain-Barré syndrome (GBS) (1361 pg/ml) had higher levels than CP (324 pg/ml, p<0.0001 and p=0.001). In addition ALS had higher levels than patients with Alzheimer’s disease (AD) (1361 pg/ml) and GBS (p<0.0001, respectively). Geometric mean and 95% CI are displayed. Dots represent individual samples. P-values are adjusted for age and corrected by Bonferroni method.

Similar to the serum results, CSF levels of NfL correlated with age in GBS (r=0.65, p=0.002) and ALS (r=0.30, p=0.048). After correction for age, a significant difference remained between GBS (p=0.001) and ALS (p<0.0001), but not AD (p=1.0) versus CP. Similarly, we confirmed the higher levels in ALS as compared to AD and GBS (p<0.0001, for both comparisons) (Figure 4).

#### C. CSF – serum relationship

Overall geometric mean levels in CSF (1,142 pg/ml, 906-1,439) were 96.8-fold higher than in serum (11.8 pg/ml, 8.5-16.5, p<0.0001; fold-increase in CSF versus serum: CP: 73.6, AD: 45.3, GBS: 17.1, ALS: 57.8, p<0.0001, respectively).

**Figure 5:**
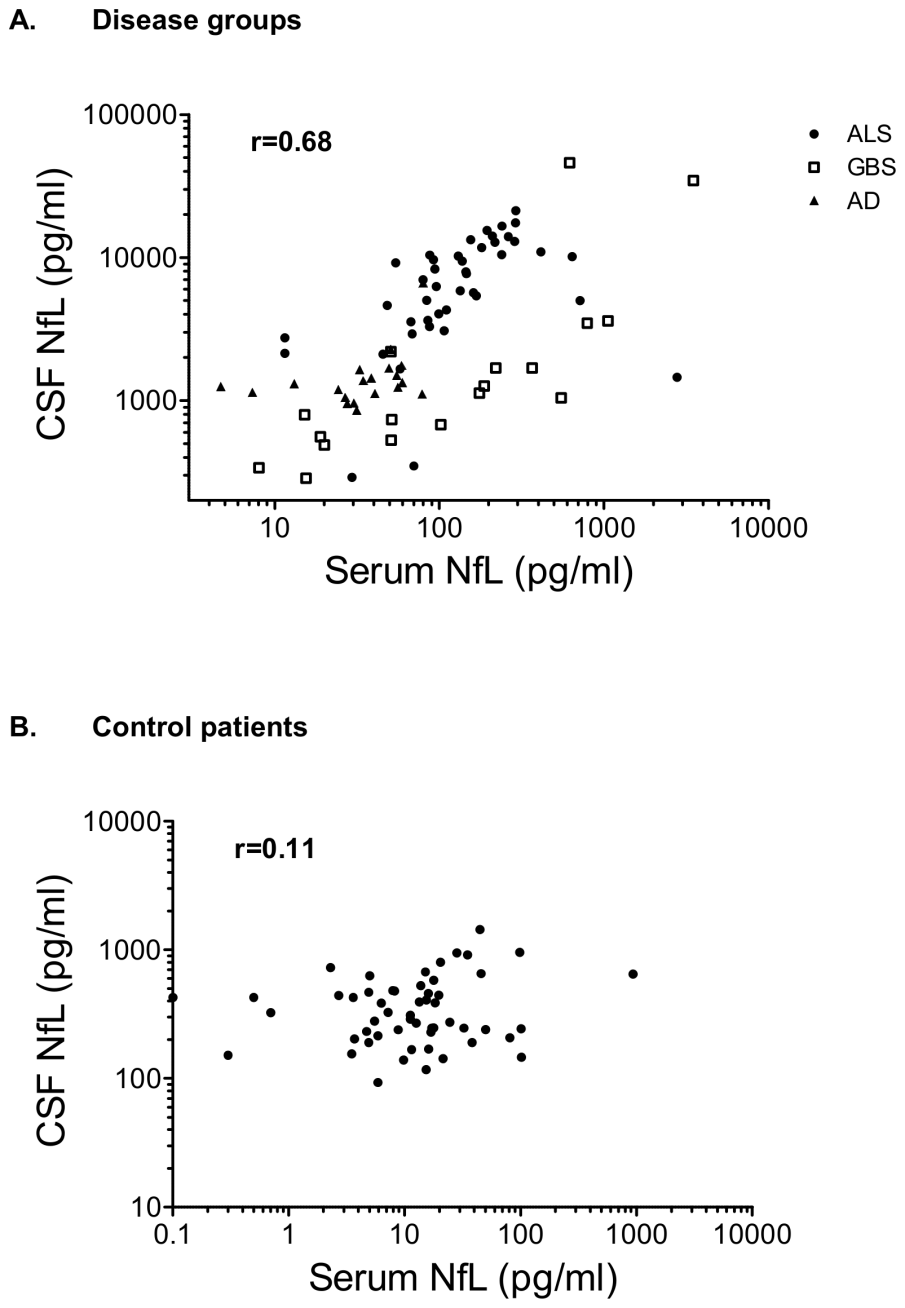
Correlation of serum and CSF NfL measurements. Serum and CSF measurements of NfL correlated in the disease groups (A): Alzheimer’s disease (AD) (r=0.48, p=0.033), Guillain-Barré syndrome (GBS) (r=0.79, p<0.0001) and Amyotrophic lateral sclerosis (ALS) (r=0.70, p<0.0001): overall: r=0.68, p<0.001. Conversely this was not seen in the control patients (CP) (r=0.11, p=0.3739) (B).

Serum and CSF measurements of NfL correlated in the disease groups (Figure 5a): AD (r=0.48, p=0.033), GBS (r=0.79, p<0.0001) and ALS (r=0.70, p<0.0001), conversely this was not seen in CP (r=0.11, p=0.3739) (Figure 5b).

## Discussion

A highly sensitive method for the detection of a clinically relevant biomarker of neurodegeneration has been developed. Importantly, our method allows us to make use of readily available longitudinal patient blood samples, instead of being restricted to ethically difficult to obtain CSF samples. One potential clinical application for serum NfL levels is demonstrated by the diagnostic sensitivity of 91.3% for ALS, a rapidly progressive neurodegenerative disease [29,30].

We present the first ECL based solid phase immunoassay for the NfL protein in blood based on two non-competitive, monoclonal antibodies. These antibodies have been widely used and validated in a commercial ELISA for CSF measurements of NfL (NF-light^®^ assay) [5,24,31]. NfL is considered to represent the most abundant and also most soluble Nf subunit [1].

The optimised ECL-NfL assay protocol proved to be highly accurate (intra-assay CV < 6%, inter-assay CV < 24%), sensitive (sensitivity 15.6 pg/ml) and demonstrated linearity and parallelism (Figures 1 and 2) over a wide analytical range (15.6-10,000 pg/ml). In addition we found NfL^Umea47:3^ to be stable in serum [25]. This is relevant for a potential value to monitor drug effects by serum NfL in ALS where Nf aggregate formation is a key pathological finding [28]. In contrast to NfH^SMI34^ and NfH^SMI35^, no such aggregates were found for NfL^Umea47:3^, essentially overcoming the limitations of the Nf “hook effect” (matrix effect) [28]. In this context a more than 20-fold elevation of serum NfL^Umea47:3^ levels in ALS compared to HC cannot be overestimated. Interestingly, the fold-differences between disease groups and CP for serum NfL^Umea47:3^ was higher compared to the respective CSF levels (serum/CSF: ALS: 21.7/17.0; AD: 7.0/4.3 and GBS: 18.0/4,2).

An important and unresolved question is whether or not there is a relevant correlation between Nf levels and age. If present, such a relationship would require age dependent cut-off values [7]. A major limitation to all studies in this field to date [6,7,11,32–34] is that they have not been powered to investigate this potential correlation in the CSF, due to lack of samples from a sufficiently large healthy control group across all age categories. Again, the availability of the present method to investigate this in readily available serum samples is highly relevant. Importantly, we did not find a correlation between serum NfL^Umea47:3^ levels and age in either HC or CP. Whether or not a possible relationship with age exists for ALS, GBS or AD is questionable, as older patients are often more severely affected and higher age is the most important prognostic factor in either condition and therefore not independent of the neurodegeneration related release of NfL^Umea47:3^.

The absence of the Nf hook-effect is an important analytical advantage for quantification of the ECL based serum NfL^Umea47:3^ assay compared to the serum NfH^SMI34^ and NfH^SMI35^ ELISA, as there is no necessity for a time-consuming pre-incubation step with urea [28]. Given the important prognostic information that NfH levels provide on a number of clinical conditions, we anticipate NfL^Umea47:3^ to be relevant for future studies. Serum NfL^Umea47:3^ bears the potential for predicting disease progression in ALS [15,35,36] and MS [17,18], detecting particularly disabling acute episodes of optic neuritis or relapses in MS [16], identifying primary and secondary brain damage in stroke [22,37], SAH [13], TBI [19,38] and in the emerging concept of chronic traumatic encephalopathy (CTE) [20,38]. Like serum NfH^SMI35^, serum NfL^Umea47:3^ may also be exploited as a safety biomarker for recognising neurotoxicity [21]. There is already data that serum NfL levels are of comparable prognostic value to NfH^SMI35^ levels following cardiac arrest [19,23]. Of note there were no controls and no analytical validation data from the NfL assay used in one study [23].

Similar to our previous findings for NfH^SMI35^ in CSF, a bimodal distribution of serum NfL levels was seen in patients with GBS [6]. There are no previous studies on Nf in blood from patients with GBS. We have earlier shown that CSF levels of NfH are higher in patients with evidence of axonal damage compared to those with purely demyelinating GBS, with CSF NfH levels predictive of outcome [9,39]. Future prospective studies incorporating detailed longitudinal clinical and electrophysiological assessments, and sampling are clearly warranted. These studies will also shed light on the role of proximal versus more distal axonotmesis and secondary axonal peripheral degeneration and the relationship of increased blood NfL levels [40].

Blood levels of Nf have similarly not been investigated in patients with dementia. In our study the differences in serum and CSF NfL levels in AD compared to HC and CP (p<0.0001 and p=0.002) lost significance after age and Bonferroni correction. This is in line with previous investigations where CSF NfH^SMI35^ levels were increased, but diagnostic sensitivity, and hence potential for clinical use of NfH ^SMI35^ was not superior to that of the benchmark biomarkers total tau, phospho tau, or amyloid beta 1-42 [41,42]. To explore these questions further we are currently expanding our database in a larger and well characterised cohort of AD and control patients.

In summary, we developed and validated a sensitive and reliable assay for measurements of NfL in human blood samples. For the first time, we were able to demonstrate increased blood NfL levels in patients with ALS and GBS. These differences were more pronounced for the ECL-NfL^Umea 47:3^ assay than those reported in ALS for NfH in previous reports [15,36]. Our data support further studies of serum NfL in well-defined longitudinal cohorts of neurodegenerative diseases. These studies will show if serum NfL measurements can be used as a biomarker for disease progression and as an outcome measure in clinical trials.
